# Emergence of Methicillin-Resistant *Staphylococcus aureus* ST239/241 *SCCmec*-III Mercury in Eastern Algeria

**DOI:** 10.3390/pathogens10111503

**Published:** 2021-11-18

**Authors:** Hanane Aouati, Linda Hadjadj, Farida Aouati, Amir Agabou, Mariem Ben Khedher, Hacène Bousseboua, Chafia Bentchouala, Jean-Marc Rolain, Seydina M. Diene

**Affiliations:** 1 Département de Microbiologie, Faculté des Sciences de la Nature et de la Vie, Université des Frères Mentouri Constantine 1, Constantine 25017, Algeria; aouati.hanane@yahoo.com; 2 MEPHI, IRD, APHM, IHU-Méditerranée Infection, Faculté de Pharmacie, Aix-Marseille Université, 13005 Marseille, France; linda.hadjadj@univ-amu.fr (L.H.); benkhedhermaryem044@gmail.com (M.B.K.); jean-marc.rolain@univ-amu.fr (J.-M.R.); 3 Faculté de Médecine, Université Salah Boubnider Constantine 3, Centre Hospitalo-Universitaire Ben Badis, Service de Microbiologie, BP 125, Constantine 25000, Algeria; c.bentchouala@yahoo.fr; 4 Département d’Anesthésie-Réanimation Chirurgicale, Université Paris Nord, APHP, Hôpital Beaujon, 92110 Clichy, France; farida.aouati@gmail.com; 5 Institut Vétérinaire, Université des frères Mentouri Constantine 1, Laboratoire de Recherche PADESCA, Constantine 25071, Algeria; amirveto@gmail.com; 6 Ecole de Biotechnologie, Université Salah Boubnider Constantine 3, Laboratoire de Génie Microbiologique, BP E66, Constantine 25000, Algeria; h.bousseboua@yahoo.fr

**Keywords:** HA-MRSA, MDR, *SCCmec* cassettes, *SCCmec*-III mercury-*ccrC*, ST239/241, *SCCmec*-V, ST34, genome sequencing HA-MRSA

## Abstract

In this paper, we investigate the epidemiology of infections-associated *Staphylococcus* *aureus* (*S. aureus*) from the Medical Intensive Care Unit (MICU) at University Hospital Center of Constantine (UHCC) in Algeria, with a special emphasis on methicillin-resistant strains (MRSA) revealed by cefoxitin disks (30 μg), then confirmed by penicillin-binding protein (PBP2a) agglutination and real-time polymerase chain reaction (RT-PCR) targeting *mecA* and *mec*C genes. Staphylococcal Cassette Chromosome *mec* (*SCCmec* type), staphylococcal protein A (*spa*-type), multilocus sequence type (MLST), Panton–Valentine Leucocidin (PVL), and toxic shock syndrome toxin-1 (TSST-1) were further investigated in all isolates, and whole genome sequencing was performed for a selected subset of three hospital-acquired MRSA (HA-MRSA) isolates. A measurement of 80% out of the 50 *S. aureus* isolates were identified as HA-MRSA harbouring the *mecA* gene, and 72.5% of them were multidrug resistant (MDR). Twelve STs, four different *SCCmec* cassettes, fourteen *spa* types, ten isolates Panton–Valentine Leukocidin (PVL)-positive, and three isolates TSST-1 were identified. Interestingly, there was a high prevalence (n = 29; 72.5%) of a worrisome emerging clone: the HA-MRSA ST239/241 *SCCme*c-III mercury with PVL negative, resistant to β-lactams, aminoglycosides, quinolones, and tetracyclines. Other clones of HA-MRSA isolates were also identified, including PVL-positive ST80 *SCCmec*-IV/*SCCmec*-unknown (22.5%), ST34 *SCCmec*-V with TSST-1 positive (2.5%), and PVL-negative ST72 *SCCmec*-II (2.5%). Genome analysis enables us to describe the first detection of both PVL-negative HA-MRSA ST239/241 SCCmec-III mercury carrying *ccrC*, as well as *SCCmec*-V cassette, which dramatically changes the epidemiology of *S. aureus* infections in one of the hospitals in eastern Algeria.

## 1. Introduction

*Staphylococcus aureus* (*S. aureus*) is one of the leading bacteria that cause skin and soft tissues infections, bacteraemia, endocarditis, osteomyelitis, pneumonia, urinary tract infections, toxic shock syndrome and bloodstreams infections [1,2]. Methicillin-resistant *S. aureus* (MRSA) isolates have been involved in healthcare-associated infections (HA-MRSA) since the 1960s and community-associated infections (CA-MRSA) since the end of the 1980s [3]. Nowadays, treatment of *S. aureus* infections has become more challenging due to (i) the widespread and rapid dissemination of MRSA isolates in both community and hospital settings and (ii) the increasing prevalence of highly multi-drug resistant (MDR) MRSA, particularly in hospitalised patients [4,5]. Methicillin resistance is mediated by the expression of an altered penicillin-binding protein (PBP2′ or PBP2a) encoded by methicillin-resistant gene variants (i.e., *mecA, mecC*) carried on the Staphylococcal Cassette Chromosome *mec* (*SCCmec*) [6,7,8]. Thus far, fourteen *SCCmec* cassettes (*SCCmec-*I to XIV) have been described on the basis of their structural organisation, their genetic content, and the combination of the *mec* and *ccr* (cassette chromosome recombinase) gene complexes [9,10,11,12]. Five of these mobile elements (*SCCmec*-I, II, III, IV and V) are the most-reported cassettes in MRSA isolates in the world [13]. *SCCmec* types I, II, and III are more frequently described in hospital-acquired (HA)-MRSA isolates, whereas community-acquired (CA)-MRSA isolates are more characterised by *SCCmec* types IV or V [14,15]. Interestingly, it has been reported that *S. aureus* isolates acquired *SCCmec* cassettes from coagulase-negative staphylococci (CoNS), especially *Staphylococcus sciuri* (*S. sciuri*) [16]. These CoNS harbour a high variety of *SCCmec* cassettes and can serve as a potential reservoir for *SCCmec* elements [17,18].

To elucidate the distribution and the epidemiology of *S. aureus*, the genotyping approach is predominantly performed based on staphylococcal protein A (*spa*) typing and multilocus sequence typing (MLST) [19]. However, molecular characterisation of *SCCmec* cassettes is also used for typing *S. aureus* isolates and for differentiating HA-MRSA from CA-MRSA isolates and their respective epidemiological origins [20]. In addition to antimicrobial resistance elements, the pathogenicity of *S. aureus* isolates is also related to the expression of various virulence genes encoding Panton Valentine Leukocidin (PVL) and toxic shock syndrome toxin-1 (TSST-1), among others. These genes can also serve as additional genetic markers in MRSA isolates characterisation [21,22]. 

Compared to other regions of the world, epidemiological studies published on MRSA isolates in North Africa are quite scarce [23] in spite of some investigations reporting the spread of MRSA isolates in Algerian hospitals. With the exception of the study by Basset et al. in 2015 describing the detection of *SCCmec-*III mercury-positive MRSA isolates identified as the typically nosocomial Brazilian clone ST239 at Bologhine Ibn Ziri University Hospital in Center of Algeria [24], no epidemiological studies have been carried out on HA-MRSA isolates harbouring this genetic element elsewhere in the country (especially in the east). Moreover, the work of Ouchenane et al. on MRSA at the Military Hospital of Constantine (from 2005 to 2007) did not present any complementary molecular data to really demonstrate the carriage of *SCCmec*-V [25]. Thus, we aim in this study to conduct an exhaustive epidemiological investigation on *S. aureus* isolates collected over two years from patients hospitalised in the Medical Intensive Care Unit (MICU) at University Hospital Center of Constantine (UHCC), Algeria (Eastern Algeria), through their prevalence, determination, and molecular characterisation with whole genome sequencing to investigate the clonality, the virulomes, and the resistomes of HA-MRSA.

## 2. Results

### 2.1. Clinical Isolates

As presented in Table 1, 50 clinical isolates of *S. aureus* were successfully collected from patients with various infections. Blood from septicaemia cases was the main sample (44%; n = 22), followed by endotracheal intubation (40%; n = 20), central venous catheter, chest drain and urine (4%; n = 2), and, finally, pleural and urinary catheter (2%; n = 1 for each one). A measurement of 54% (n = 27) of the specimens was collected from women and the remaining 38% (n = 19) from men. All patients were between the ages of 2 and 84, and they were classified into nine age groups, with the age group 41–50 years old (16%; n = 8) being the most infected by *S. aureus*.

### 2.2. Antibiotic Susceptibility

As shown in Table 2, the highest level of antibiotic resistance was observed against methicillin (80%; n = 40 HA-MRSA). For these HA-MRSA isolates, a high prevalence (97.5%; n = 39) of penicillin and co-resistance to kanamycin was recorded, followed by tobramycin (75%; n = 30). A measurement of 72.5% (n = 29) of all isolates was resistant to gentamicin, ciprofloxacin, ofloxacin, and tetracycline. Erythromycin resistance was observed in 35% (n = 14) of the isolates, whereas the lowest resistance rate was shown for fusidic acid and trimethoprim-sulfamethoxazole with 12.5% (n = 5) each and clindamycin with 5% (n = 2). No resistance was noticed against vancomycin, teicoplanin, linezolid, mupirocin, rifampicin, pristinamycin, fosfomycin, and tigecycline. A high rate of HA-MRSA isolates (72.5%; n = 29) exhibited multidrug resistance phenotypes (*p* < 0.001) (Table 2).

Only 20% (n = 10) of all isolates were HA-MSSA, and they were susceptible to almost all tested antibiotics, with the exception of penicillin (100% resistance), erythromycin (30% resistance; n = 3), tetracycline (20% resistance; n = 2). Only 10% (n = 1) of them were less susceptible to both clindamycin and fusidic acid.

**Figure 1:**
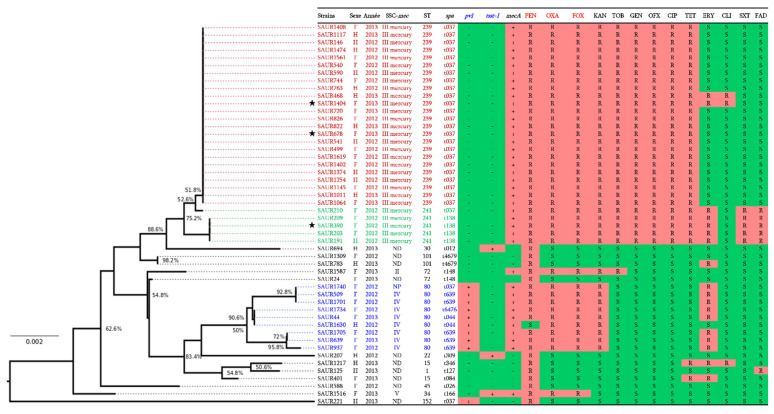
Phylogenetic tree of 50 *S. aureus* clinical isolates from MICU at the UHC of Constantine (Algeria) based on MLST-concatenated gene sequences of each isolate shown. The most prevalent strains are HA-MRSA ST239/241 harbouring *SCCmec*-III mercury, without the presence of the PVL gene and high multidrug resistance to different antibiotics. SAUR: *Staphylococcus aureus*, PEN: penicillin, OXA: oxacillin, FOX: cefoxitin, KAN: kanamycin, TOB: tobramycin, GEN: gentamicin, OFX: ofloxacin, CIP: ciprofloxacin, TET: tetracycline, ERY: erythromycin, CLI: clindamycin, SXT: trimethoprim/sulfamethoxazole, FAD: fusidic acid, NP: not performed, ND: not determined. The three isolates indicated by asterisk are those selected and subjected to whole genome sequencing.

Seven resistance phenotypes were observed in MRSA isolates, with the phenotype penicillin-kanamycin-tobramycin-gentamicin-ciprofloxacin-ofloxacin-tetracycline being the most prevalent (55%; n = 22; *p* = 0.001) (Figure 1). The statistical analysis could not prove any significant impact or correlation between the nature of samples, the sex/age of the patients, and the prevalence of MDR strains.

### 2.3. Molecular Characterisation

As shown in Figure 1, none of the 40 HA-MRSA clinical isolates harboured the *mecC* gene, and the *mecA* gene was detected in all of them. *SCCmec* typing revealed that both *SCCmec*-III and I were absent in all isolates; however, 72.5% (n = 29) of them were confirmed to harbour the *SCCmec*-III mercury cassette. The presence of this cassette type was significantly higher (*p* < 0.001), followed by *SCCmec*-IV cassette (20%; n = 8), and the remaining isolates carried the types II and V and an unknown *SCCmec* cassette (2.5%; n = 1 for each one). 

Interestingly, co-resistance to β-lactams, aminoglycosides, fluoroquinolones, and tetracyclines was more established among the *S. aureus* isolates harbouring the *SCCmec*-III mercury cassette with a high prevalence of 75.9% (*p* < 0.001). All HA-MRSA strains with an MDR phenotype harboured the *SCCmec*-III mercury (*p* < 0.001). Inversely, the *SCCmec*-IV cassette was only identified among strains resistant to β-lactams, to kanamycin, and to erythromycin.

The PVL toxin gene was carried by nine (22.5%) out of the forty HA-MRSA isolates and was associated with ST80. Furthermore, only one HA-MRSA related to an ST34 isolate tested positive for the TSST-1 toxin gene. The prevalence of PVL genes was low in HA-MRSA isolates with *SCCmec*-IV and the unknown *SCCmec* type (20%; n = 8 and 2.5%; n = 1, respectively). Remarkably, no isolates with *SCCmec*-II, *SCCmec*-III mercury tested positive for PVL toxin genes. MLST and *spa* typing revealed five STs, including ST239 (60%; n = 24), ST241 (12.5%; n = 5), ST34 (2.5%; n = 1), ST80 (22.5%; n = 9), and ST72 (2.5%; n = 1) and seven *spa* types, including t037, t138, t166, t639, t6476, t044, and t146. The ST239/241-negative *pvl* was the most predominant ST and was significantly associated with the *spa* type t037 and the *SCCmec*-III mercury (60%; n = 24). The ST80 was related to positive PVL genes with different *spa* types t639 (12.5%; n = 5), t044 (5%; n = 2), and t6476 (2.5%; n = 1) for *SCCmec*-IV and t037 (2.5%; n = 1) for the unknown *SCCmec* cassettes. Conversely, the single ST72 clone identified in this study harbours the *SCCmec*-II cassette and *spa* t148 without the *pvl* and TSST-1 genes.

The only TSST-1 gene detected in this study was found in a *SCCmec*-V HA-MRSA strain of ST34 with t166 (Figure 1).

### 2.4. Genomic Analysis of SCCmec Cassettes from Sequenced Genomes

**Figure 2:**
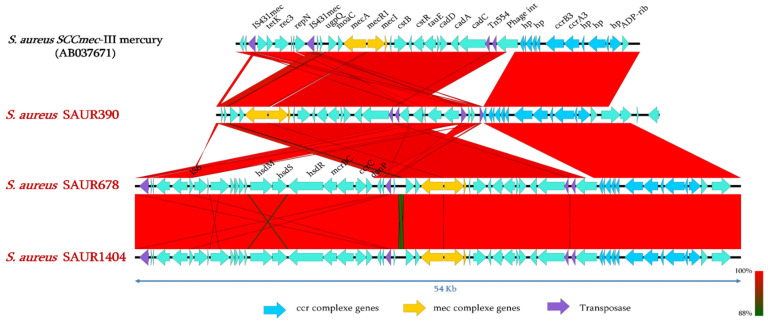
Comparison of the genetic structures of *SCCmec* elements from *S. aureus* strains 85/2082 (AB037671.1), SAUR390, SAUR678, and SAUR1404. The arrows indicate the positions and directions of the *orfs*. Regions of more than 88% homology, determinates by Blastn, are marked in red. Insertion sequences are annotated (purple), *ccr* complex genes (blue), and *mec* complex genes (yellow). The predicted function of each gene name is presented inSupplementary Materials Table S1.

To investigate the genetic structure of the detected *SCCmec* cassettes of our isolates, three of them (SAUR390, SAUR678, and SAUR1404, marked with a star in Figure 1) were sequenced using Illumina Miseq. The genome assembly resulted in genome sizes of 2′920′844-bp, 2′903′748-bp, and 2′853′533-bp assembled into 124 contigs, 147 contigs, and 177 contigs for SAUR390, SAUR678, and SAUR1404 isolates, respectively. As shown in Figure 2, sequence comparison with the *SCCmec* cassettes between our isolates and that of *SCCmec*-III mercury reveals high similarity (≥80% nucleotide identity), although some recombinations are identified. Interestingly, we can observe an integration of a complete Type I restriction-modification system (RMS) within the *SCCmec* of SAUR678 and SAUR1404 (Figure 2).

### 2.5. Resistance Genes Analysis of HA-MRSA SCCmec-III Mercury

As shown in Table 3 and Supplementary Materials Table S2, antibiotic resistance genes were identified across the chromosomes and the plasmids of the three sequenced genomes. These included β-lactams resistance genes *mecA (methicillin)* and *blaZ (penicillin)*; aminoglycosides resistance genes *ant*(9)-Ia, (*agly*) *aph*Stph, (*agly*)*aad*C, *aad*(6), *aph*(3′)-IIIa, aac(6′)-Ie-*aph*(2′’)-Ia, *aph*(3′’)-Ib, *aph*(6)-Id, and *aph*(3′)-Ia; streptothricin resistance gene sat-4; tetracycline resistance genes *tet*(38), *tet(K), and* *tetM*; *sulfamid resistance genes dfrC, dfrE, and dfrG;* macrolids-lincosamides-streptogramin B antibiotic (MLS_B_) resistance genes ermA and ermC; phenicols resistance gene (phe)dha1; quinolone resistance genes norA, norB, mgrA, arlR, and arlS; fosfomycin resistance gene fosB3; and other antibiotic resistance genes, such as elfamycin ef-tu, which confer resistance to elfamycin antibiotics. This identification correlated with the results of antibiotic susceptibility testing, as the strains were found to be resistant to 13 out of 21 antibiotics tested, but were susceptible to vancomycin, teicoplanin, linezolid, mupirocin, rifampicin, pristinamycin, fosfomycin, and tigecycline. Among the identified resistance genes, *blaZ*, *aph-*Stph, aac(6′)-Ie-*aph*(2′’)-Ia, *norA*, *norB*, *mgrA, arlR, arlS,* *tet*(38), *tet*(k), and *tetM* were the most prevalent (29/29; 100%), followed by *ermA* (7/29; 24.1%). 

### 2.6. Virulence Genes in HA-MRSA SCCmec-III Mercury

As presented in Table 4 and (Supplementary Materials Table S3), various virulence genes were also identified in the three sequenced genomes. All of the sequenced strains harboured genes encoding immunomodulators (*spa*, *isdA*, *isdB*, *isdC*, *isdD*, *isdE*, *isdF*, *isdG*, *sbi*, *esaA*, *esaB*, *essA*, *essB*, *essC,* and *esxA*) (29/29; 100%), as well as different adhesins (*lap*, *sdrC*, *sdrD*, *sdrE*, *clfA*, *clfB*, *fnbA*, *fnbB*, *psaA*, *vwbp*, *ebp*, *map*, *icaA*, *icaB*, *icaC*, *icaD*, *icaR,* and *cna*) (29/29; 100%). Genes encoding exoenzymes, such as *ads*A (adenosine synthase A), coa (staphylocoagulase), *geh* and *lip* (lipase), *srtB* (sortase B), *katA* (catalase), *sspA*, *sspB*, *sspC*, *clpC*, *clpE* and *clpP* (protease), *aur* (zinc metalloproteinase aureolysin), *ureB* and *areG* (urease), *hysA* (hyaluronate lyase), *cpsA* and *cpsJ* (polysaccharide capsule synthesis protein), and *htpB* (heat shock protein), were present in all isolates (29/29; 100%). Many isolates had a staphylokinase precursor gene *(sak)* (24/29; 82.7%) with the complement inhibitor *gene* (*scn*) (24/29; 82.7%) being the most prevalent. With the exception of capsular polysaccharide synthesis enzyme genes, such as *cap8A*, *cap8B*, and *cap8C* genes, which were present in a small portion of these isolates (5/29; 17.2%), no other *cap8* genes (D to P) were identified for all isolates. In addition, different toxins-encoding genes were identified in these isolates. Haemolysins-encoding genes (*hlb*, *hld*, *hlgA*, *hlgB,* and *hlgC*) were present in all isolates (29/29; 100%). The *lukF-PV* gene encoding Panton Valentine Leukocidin component F was also present in all isolates (29/29; 100%). However, 24 of them had the enterotoxin genes *sea*, *selk,* and *selq* (24/29; 82.7%).

## 3. Discussion

In this epidemiological study, as part of the first molecular and genomic investigation of *S. aureus* healthcare-associated infections in MICU, our findings reveal a worrisome emergence with a high prevalence (72.5%) of HA-MRSA ST239/241-t037/138 isolates carrying PVL-negative *SCCmec*-III mercury element, responsible for healthcare-associated infections in the Constantine hospital (north-eastern Algeria). The high prevalence of HA-MRSA isolates we have recorded (80%) strongly corroborates earlier Algerian reports describing a prevalence of 75% in 2011 and 71.4% in 2014 at the cities of Tlemcen and Annaba, respectively [26,27]. This prevalence seems to have increased since the first study, rising from 33.2% in 2006 at one hospital in Algiers [28] to 80% in our recent investigation. Furthermore, it remains higher than those reported in European countries, including Italy (29%), Greece (27.3%), Romania (21.7%), Spain (12.5%), Germany (8.3%), France (7%), and the UK (0%) [29]. 

Most of our HA-MRSA isolates (42.5%) were recovered from the bloodstream of patients aged 41 to 50, most of whom were female [30], which again proves that HA-MRSA is one of the leading causes of bloodstream infections in MICU. In addition, our findings reveal significant spread diffusion of multidrug resistance among HA-MRSA isolates harbouring the *SCCmec-*III mercury element, which limits the options for effective antibiotic treatment of the infections due to these isolates. Inversely, previous results from a hospital setting in Algiers indicated that HA-MRSA strains harbouring the SCC*mec*-III mercury element are less prevalent and only few (28.6%) of them are MDR [24]. None of our MSSA isolates were MDR, which is similar to previously published results from Algeria [31]. On the other hand and in line with most previous reports in Algeria [32,33,34], our *S. aureus* isolates were entirely susceptible to vancomycin.

*SCCmec* types I, II, and III are mostly reported in HA-MRSA isolates, while *SCCmec*-IV and V are related to CA-MRSA [9]. The Brazilian MRSA clone (ST239, *SCCmec*-III, and PVL negative) has been reported in various countries [23,35]. Therefore, it seems that the prevalence of the Brazilian clone linked to the variant *SCCmec*-III mercury element in MICU at Constantine hospital is high (72.5%) in HA-MRSA isolates without the presence of PVL genes. In our study, which focused on the genetic characterisation of HA-MRSA isolates, the presence of *S. aureus* isolates harbouring different *SCCmec*-III mercury, *spa* types, and ST(s) has been attested. This is the first detection of ST239 and ST241 HA-MRSA isolates with *SCCmec*-III mercury and *spa* t037 and t138 in eastern Algeria. These ST239/ST241 SCC*mec*-III mercury clones are predominant (72.5%) with a highly significant prevalence of multi-resistance phenotypes (55%) (*p <* 0.001). As compared to reports from central Algeria, only 8.3% of HA-MRSA carried the SCC*mec*-III mercury element [24]. However, HA-MRSA isolates carrying SCC*mec*-V have been reported worldwide [36,37] and also in another hospital in the city of Constantine by no thorough molecular analysis [25,38]. Advanced analyses of the sequencing genome in our investigation improved our understanding of the structural organisation of the SCC*mec* cassettes. Therefore, our study has all the advantages of confirming the first detection of SCC*mec*-V, which is TSST-1 gene positive. We report here the detection of ST34 HA-MRSA isolates with SCC*mec*-V and *spa* t166, which had previously only been detected in MSSA strains in Africa [39,40]. The presence of this clone carrying *SCCmec*-V may be due to horizontal gene transfer [41].

*SCCmec*-IV, ST80 has been reported in both community-acquired and hospital-acquired infections in Algerian hospitals [24,34]. Patients in this MICU suffer from infections caused by 20% of HA-MRSA carrying the SCC*mec* type IV, and our own findings confirmed the presence of the MRSA-IV European clone.

Analysis of drug resistance and virulence genes revealed evidence of a great diversity in their combinations among our characterised strains. In general, HA-MRSA strains are commonly multidrug resistant [42], harbour *SCCmec* type II and III, and demonstrate multiresistance, containing additional antibiotic resistance genes, such as erythromycin and tetracycline, as well as methicillin. The SCC*mec*-III element confers resistance to a broad spectrum of antibiotics and heavy metals [43]. The multi-resistant nature of our MRSA clones could be explained by the presence of several resistance genes in the SCC*mec* cassettes, as previously described [44]. As reported by Soliman *et al.* (2020) [45] our strains harboured both *mec*A and *bla*Z encoding resistance to β-lactams. Several studies have mentioned a concordance between phenotypic and genotypic resistance results in MRSA strains [46,47], which is in perfect agreement with our findings. Rahimi et al. (2014) [48] reported that *ermB* was prevalent in just a few erythromycin-resistant staphylococci, and in the study by Abbassi et al. (2017) [49], this gene was not identified in HA-MRSA. The resistance to erythromycin in these studies was due to the carriage of *ermA* and *ermC* by MRSA strains in Iran. This aligns well with our findings, since *ermB* plays no role in macrolide-resistant HA-MRSA. The multi-drug resistant patterns for the HA-MRSA in our study are in keeping with what has been described in other countries in Africa. According to Bendary et al. (2020) [50], MDR HA-MRSA ST239/241 strains are less toxicogenic. This may be due to the acquisition of toxin genes on mobile elements to the detriment of extended antibiotic resistance [51]. As in our study, HA-MRSA ST239/241 strains with very low-level resistance to glycopeptides have been reported in many other countries [52,53], which proves that they are among the most effective drugs against infections due to this clone. Regarding resistance to fluoroquinolones, Japoni et al. (2011) [54] observed that SCC*mec* III isolates showed high rates of resistance to ciprofloxacin. This concurs well with our findings concerning the variant *SCCmec*-III mercury, as we detected many genes encoding resistance to these antibiotics among our isolates.

The wide collection of virulence factors harboured by *S. aureus* are differently present within clones [55,56] in relation to the high variety of infections for which they are responsible. PVL is a crucial cytotoxic virulence factor in serious infections (necrotising pneumonia, skin and soft tissue infections) [57,58]. It is widely described as the primary virulence factor driving the epidemic spread of the European clone [59]. This corroborates our results, as we found, for instance, ST80 to be PVL*-*positive and ST239/241 to be PVL-negative.

Several enterotoxin genes can be harboured by enterotoxigenic *S. aureus* strains [60]. Toxinogenic strains may pose a public health risk to consumers, as they can contaminate food and cause food poisoning. The TSST-1-positive MRSA strains are highly virulent and provoke a variety of health disorders, including toxic shock syndrome and various suppurative infections. In addition, the toxic shock syndrome toxin (TSST-1) and staphylococcal enterotoxins are reported to play a significant role in the proliferation of T cells, irrespective of antigenic specificity [61,62].

## 4. Conclusions

Due to the critical situation of patients admitted in to intensive care and given the high prevalence of HA-MRSA isolates described in Algeria, our study confirms that these findings in the MICU at the UHCC are linked to a new molecular and epidemiological change of HA-MRSA following the first detection of the ST239/241 *SCCmec*-III mercury and ST34 *SCCmec*-V clones. The first is expressed by high frequency and is significantly associated with multi-drug resistance to the antimicrobial agents used to treat patients in intensive care. This implies a serious threat in hospital settings, as well as in the community, requiring adequate control measures to prevent the spread, transmission dynamics, and epidemics of these clones. Proper management of antimicrobial agents and a better awareness among healthcare personnel is therefore needed to combat these nosocomial MRSA infections. In addition, other studies are needed to screen for CA-MRSA isolates based on isolation and decolonisation upon admission to evaluate the HA-MRSA-IV clone reservoir. The rapid MRSA diagnostic methods used in this investigation should be established to monitor and implement the successful treatment of these MRSA nosocomial infections.

## 5. Materials and Methods

### 5.1. Isolates and Identification

From January 2012 to December 2013, non-duplicate *S. aureus* isolates were collected from MICU UHCC in the east of Algeria. All *S. aureus* isolates were cultured from clinical samples, including from the bloodstream, tracheal, drain, urine, pleural and urinary catheters, in infection contexts with or without the presence of polynuclear cells in the biological samples. HA-MRSA isolates are defined as isolates recovered from hospitalised patients 48 h after admission presenting resistance to methicillin. Demographic characteristics and clinical data were collected from the patient records. All isolates were grown on Chapman and Colombia blood agar media for 24 h at 37 °C. Isolates were pre-emptively identified using conventional methods. The purity of the *S. aureus* isolates transported was checked using a Trypticase Soja Agar medium at the IRD, APHM, MEPHI, IHU–Méditerranée Infection (Marseille, France), where their molecular identification was confirmed using matrix-assisted laser desorption–ionization time of flight mass spectrometry Microflex (MALDI-TOF-MS) (Bruker Daltonics, Bremen, Germany) as described [63]. The pure identified *S. aureus* strains were conserved in a storage medium for further molecular characterisation.

### 5.2. Antimicrobial Susceptibility Testing

Susceptibility testing against 21 antibiotics was performed using the Kirby-Bauer disk diffusion method on Mueller-Hinton (MH) agar. The results were interpreted according to the Clinical Laboratory Standard Institute (CLSI) guidelines [64], with the exception of fusidic acid, for which we used the cut-off values of the European Committee on Antimicrobial Susceptibility Testing (EUCAST) [65]. MRSA isolates were identified using cefoxitin disks [66], in addition to (i) a growth test on MH agar supplemented with 4% NaCl and 6 µg/mL of oxacillin, incubated in ambient air for 24 h, and (ii) an MRSA-screen latex agglutination test to detect PBP2a proteins [67]. *S. aureus* ATCC 25,923 was used as reference control strain.

### 5.3. Molecular Characterisation

Genomic DNA was extracted from bacterial cultures using a commercial DNA extraction kit (QIAGEN-Germany). Real time PCR (RT-PCR) was applied to detect the presence of *mecA* and *mecC* genes and also to investigate *SCCmec* types I to V among the isolates. All *S. aureus* isolates were also characterised by standard PCR to confirm the type of *SCCmec* cassette. The primers and probes used in this study are presented in (Supplementary Materials Table S4). The presence of PVL and TSST-1 virulence genes was checked by RT-PCR. *Spa* typing analysis was performed by DNA sequencing of repeat regions of the protein A gene for all *S. aureus* isolates, as previously described [68]. Multi-locus sequence typing (MLST) analysis of all isolates was carried out by PCR amplification and sequencing of seven house-keeping genes including *arc*, *aroE*, *glpF*, *gmk*, *pta*, *tpi,* and *yqil*, as reported [69]. Allelic profiles and sequence types (STs) were assigned according to the *S. aureus* MLST database (Available online: http://mlst.zoo.ox.ac.uk, accessed on 1 February 2017).

### 5.4. Whole Genome Sequencing (WGS)

WGS was applied to three selected HA-MRSA strains (each representative of an MDR profile) to perform comparative genomics and to match their *SCCmec* cassettes against those reported in the literature. For this, their respective purified genomic DNAs were sequenced on the Illumina MiSeq machine (Illumina, San Diego, CA, USA) using 250-bp paired-end reads and barcodes according to the TruSeq protocol and the manufacturer’s recommendations. Read quality was evaluated with the FastQC programme (Available online: http://www.bioinformatics.babraham.ac.uk/projects/fastqc/, accessed on 2 January 2018) and filtered using the Fastq-mcf programme (Ea-utils package: http://code.google.com/p/ea-utils, accessed on 2 January 2018). Genome sequences were then assembled using an A5-miseq [70] pipeline and annotated with the Prokka v1.12 programme [71]. Genes conferring antimicrobial resistance and virulence genes were investigated using the ABRICATE pipeline, which hosts different databases, including antibiotic resistance genes and virulence genes databases. The genetic environment of the *SCCmec*-III mercury element was investigated using the Easyfig version x2.2.3 programme (Available online: http://mjsull.github.io/Easyfig/, accessed on 15 June 2020).

### 5.5. Statistical Analyses

The statistical analysis was performed using the Chi-square test or Fisher’s exact test as appropriate in the IBM SPSS statistics software (V. 24.0; 2016) (C D C, Atlanta, GA, USA). Results were considered to be statistically significant at a *p* value ≤ 0.05.

## Figures and Tables

**Table 1:** Distribution of HA-MRSA and HA-MSSA according to demographic and clinical characteristics of patients in MICU-UHCC.

	All Strains	HA-MRSA	HA-MSSA
	n = 50	n = 40	n = 10
Samples			
Bloodstream	22 (44%)	17 (42.5%)	5 (50%)
Tracheal incubation	20 (40%)	15 (37.5%)	5 (50%)
Central catheter	2 (4%)	2 (5%)	-
Chest drain	2 (4%)	2 (5%)	-
Urine	2 (4%)	2 (5%)	-
Pleural	1 (2%)	1 (2.5%)	-
Urinary catheter	1 (2%)	1 (2.5%)	-
Sex			
Men	19 (38%)	13 (32.5%)	6 (60%)
Women	27 (54%)	23 (57.5%)	4 (40%)
Age (years)			
0–10	4 (8%)	4 (10%)	-
11–20	4 (8%)	4 (10%)	-
21–30	7(14%)	4 (10%)	3 (30%)
31–40	5 (10%)	3 (7.5%)	2 (20%)
41–50	8 (16%)	8 (20%)	-
51–60	3 (6%)	3 (7.5%)	-
61–70	6 (12%)	4 (10%)	2 (20%)
71–80	7 (14%)	5 (12.5%)	2 (20%)
81–90	2 (4%)	1 (2.5%)	1 (10%)

HA-MSSA: Hospital-acquired methicillin-sensitive *Staphylococcus aureus*.

**Table 2:** Distribution of resistant HA-MRSA and HA-MSSA strains isolated from patients in MICU-UHCC.

Antibiotics	HA-MRSA	HA-MSSA	Total	*p* Value
	n = 40	n = 10	n = 50	
Penicillin (10 UI)	39 (97.5%)	10 (100%)	49 (98%)	n.s
Oxacillin (1 µg)	40 (100%)	-	40 (80%)	<0.001
Cefoxitin (30 µg)	40 (100%)	-	40 (80%)	<0.001
Kanamycin (30 µg)	39 (97.5%)	-	39 (78%)	<0.001
Tobramycin (10 µg)	30 (75%)	-	30 (60%)	<0.001
Gentamicin (15 µg)	29 (72.5%)	-	29 (58%)	<0.001
Erythromycin (15 µg)	14 (35%)	3 (30%)	17 (36%)	n.s
Clindamycin (2 µg)	2 (5%)	1 (10%)	3 (6%)	n.s
Ciprofloxacin (5 µg)	29 (72.5%)	-	29 (58%)	<0.001
Ofloxacin (5 µg)	29 (72.5%)	-	29 (58%)	<0.001
Trimethoprim-sulfamethoxazole (1.25 µg, 23.75 µg)	5 (12.5%)	-	5 (10%)	n.s
Fusidic acid (30 µg)	5 (12.5%)	1 (10%)	6 (12%)	n.s
Tetracycline (15 µg)	29 (72.5%)	2 (20%)	31 (62%)	0.007
Pristinamycin (15 µg)	-	-	-	-
Tigecycline (15 µg)	-	-	-	-
Rifampicin (5 µg)	-	-	-	-
Linezolid (10 µg)	-	-	-	-
Mupirocin (5 µg)	-	-	-	-
Vancomycin (30 µg)	-	-	-	-
Teicoplanin (30 µg)	-	-	-	-
MDR	29 (72.5%)	-	29 (58%)	<0.001

*p* < 0.05 significant; n.s: not significant, HA-MSSA: Hospital-acquired methicillin sensitive *S. aureus*.

**Table 3:** Resistome analysis of the three sequenced genomes among HA-MRSA selected in MICU-UHCC. Predicted function of each gene and their % identities with described genes are given in (Supplementary Materials Table S2).

Antibiotic families	SAUR390	SAUR678	SAUR1404
Beta-lactams	*mec*A; *bla*Z; *mec*R1; *mec*I	*mec*A; *bla*Z; *mec*R1; *mec*I	*mec*A; *bla*Z; *mec*R1; *mec*I
Aminoglycosides	*ant*(9)-Ia; *aph*-Stph; *aad*C; *aph*(3′)-IIIa; *aac*(6′)-Ie-*aph*(2′’)-Ia	*aph*-Stph; aad(6); *aac*(6′)-Ie-*aph*(2′’)-Ia; *aph*(3′’)-Ib; *aph*(6)-Id; *aph*(3′)-Ia; *aph*(3′)-IIIa	*aph*-Stph; *aph*(6)-Id; *aac*(6′)-Ie-*aph*(2′’)-Ia
Streptothricins	*Sat-4*	*Sat-4*	/
Tetracyclines	*tet*(38); *tet*(K); *tet*M	*tet*(38); *tet*(K); *tet*M	*tet*(38); *tet*(K); *tet*M
Sulfamids	*dfr*C; *dfr*E; *Dfr*G	*dfr*C; *dfr*E	*dfr*C; *dfr*E
Macrolids	*erm*(C); *erm*(A)	/	*erm*(C); *erm*(A)
Phenicols	*dha*1	*dha*1	*dha*1
Quinolones	*nor*A; *nor*B; *mgr*A; *arl*R; *arl*S	*nor*A; *nor*B; *mgr*A; *arl*R; *arl*S	*nor*A; *nor*B; *mgr*A; *arl*R; *arl*S
Fosfomycin	*fos*B3	*fos*B3	*fos*B3
Efflux pumps	Three copies of sav1866; *mep*A; *mep*R	Three copies of sav1866; *mep*A; *mep*R	Three copies of sav1866; *mep*A; *mep*R

**Table 4:** Virulence genes analysis of the three sequenced genomes among HA-MRSA selected in MICU-UHCC. Predicted function of each gene and their % identities with described genes are given in (Supplementary Materials Table S3).

Virulence Factors	SAUR390	SAUR678	SAUR1404
Immunomodulators	*spa; isdA; isdB; isdC; isdD; isdE; isdF; isdG; sbi; esaA; esaB; essA; essB; essC; esxA*	*spa; isdA;isdB;isdC; isdD; isdE; isdF; isdG; sbi; esaA; esaB;essA; essB; essC; esxA*	*spa; isdA;isdB;isdC; isdD; isdE; isdF; isdG; sbi; esaA; esaB;essA; essB; essC; esxA*
Adhesins	*Lap; sdrC; sdrD; sdrE; clfA; clfB; fnbA; fnbB; psaA; vwbp; ebp; map; icaA; icaB; icaC; icaD; icaR; cna*	*Lap; sdrC; sdrD; sdrE; clfA; clfB; fnbA; fnbB; psaA; vwbp; ebp; map; icaA; icaB; icaC; icaD; icaR; cna*	*Lap; sdrC; sdrD; sdrE; clfA; clfB; fnbA; fnbB; psaA; vwbp; ebp; map; icaA; icaB; icaC; icaD; icaR; cna*
Exoenzyme	*adsA; coa; geh; lip; srtB; katA; sspA; sspB; sspC; clpC; clpE; clpP; aur; ureB; ureG; hysA; cpsA; cpsJ; htpB; cap8A; cap8B; cap8C; cap8D; cap8E; cap8F; cap8G; cap8H; cap8I; cap8J; cap8K; cap8L; cap8M; cap8N; cap8O; cap8P*	*adsA; coa; geh; lip; srtB; kat; sspA; sspB; sspC; clpC; clpE; clpP; aur; ureB; ureG; hysA; cpsA; cpsJ; htpB; sak; scn; cap8D; cap8E; cap8F; cap8G; cap8H; cap8I; cap8J; cap8K; cap8L; cap8M; cap8N; cap8O; cap8P*	*adsA; coa; geh; lip; srtB; kat; sspA; sspB; sspC; clpC; clpE; clpP; aur; ureB; ureG; hysA; cpsA; cpsJ; htpB; sak; scn; cap8D; cap8E; cap8F; cap8G; cap8H; cap8I; cap8J; cap8K; cap8L; cap8M; cap8N; cap8O; cap8P*
Toxins	*hlb; hld; hlgA; hlgB; hlgC; lukF-PV*	*hlb; hld; hlgA; hlgB; hlgC; lukF-PV; sea; selk; selq*	*hlb; hld; hlgA; hlgB; hlgC; lukF-PV: sea; selk; selq*

## Data Availability

The datasets generated during and/or analyzed during the current study can be find in the main text and the Supplementary Materials.

## Supplementary Materials

The following are available online at https://www.mdpi.com/article/10.3390/pathogens10111503/s1, Supplementary Table S1: Predicted function of genes shown in Figure 2. Supplementary Table S2: Predicted function of resistance genes presented in Table 2 and their % of identities with described genes of resistance genes. Supplementary Table S3: Predicted function of virulence genes presented in Table 3 and their % identities with described genes. Supplementary Table S4: Primers and probes used in this study for the molecular characterisation of the clinical *S. aureus* isolates from the MICU at the UHCC. Whole Genome Sequences of the three sequenced *S. aureus* isolates here are deposited at DDBJ/ENA/GenBank under the accession numbers *S. aureus* SAUR1404 (PKSS01000000), *S. aureus* SAUR678 (PKST01000000), and *S. aureus* SAUR390 (PKSU01000000).
