# Supplementary material for: Emergence of Methicillin-Resistant Staphylococcus aureus ST239/241 SCCmec-III Mercury in Eastern Algeria
Source: Pathogens. 2021 Nov 18;10(11):1503. doi: 10.3390/pathogens10111503 (PMC8621676; doi:10.3390/pathogens10111503)
Supplement: Supplementary file 1 [file pathogens-10-01503-s001.zip › Table S1.pdf]

Table S1: Predicted function of genes shown on Figure 2.

Gene name	length (bp)	%GC content	Predicted functions
ADP-rib	573	28,97	ADP-ribosyltransferase
HP	1032	27,03	hypothetical protein
HP	288	34,72	hypothetical protein
HP	1569	36,71	hypothetical protein
HP	276	32,97	hypothetical protein
recombinase	1347	34,15	casette chrs recombinase A3
recombinase	1629	35,73	casette chrs recombinase B3
HP	351	33,33	hypothetical protein
HP	90	30,00	hypothetical protein
HP	312	32,37	hypothetical protein
HP	522	32,18	hypothetical protein
HP	207	29,95	hypothetical protein
RadC	324	34,26	RadC domain protein
Phage int	1893	33,60	Tphage integrase family protein
Tn554	378	30,16	transposase
cadC	366	33,61	Cadmium resistance transcriptional regulatory protein CadC
cadA	2415	40,54	Cadmium-transporting ATPase
cadD	687	34,79	Cadmium resistance transporter
tauE	630	33,02	sulfite exporter TauE/SafE family protein
cstR	261	24,90	persulfide-sensing transcriptional repressor CstR
dsrE	1065	32,30	dihydroneopterin aldolase
cstB	939	33,12	persulfide dioxygenase-sulfurtransferase CstB
-	261	30,65	rhodanese-like domain-containing protein
roK	1149	28,72	ROK family protein
-	143	30,77	psm_mec_RNA
mecI	204	27,45	Methicillin resistance regulatory protein MecI
mecR1	1542	28,47	beta-lactam sensor/signal transducer MecR1
mecA	2007	30,59	PBP2a beta-lactam-resistant peptidoglycan transpeptidase MecA
maoC	429	29,14	MaoC family dehydratase
ugpQ	744	33,47	Glycerophosphoryl diester phosphodiesterase
HP	231	35,06	hypothetical protein
-	168	29,17	HMG-CoA synthase
IS431mec	675	34,37	IS6 family transposase IS431mec
repN	945	32,17	Replication initiation protein
-	95	33,68	ctRNA_pT181
HP	102	26,47	hypothetical protein
rec3	1242	30,19	plasmid recombination protein
MFS	549	28,96	MFS transporter
tetK	888	28,04	tetracycline efflux MFS transporter Tet(K)
IS6	357	32,49	IS6 family transposase
dnaP	1218	31,61	DNA primase
-	117	34,19	hypothetical protein
ccrC	1680	33,27	Recombinase family protein

-	339	27,73	hypothetical protein
-	90	35,56	hypothetical protein
-	312	33,65	hypothetical protein
-	504	36,11	hypothetical protein
-	222	26,58	hypothetical protein
atpase	1977	27,26	AAA family ATPase
mcrBC	1323	26,68	Putative McrBC 5-methylcytosine restriction system component
hsdR	3120	35,54	Type I restriction endonuclease subunit R
hsdS	1230	26,99	Restriction endonuclease subunit S
hsdM	1515	33,40	Type I restriction-modification system subunit M
cdnS	996	25,70	Cyclic dipyrimidine nucleotide synthase
-	549	28,96	hypothetical protein
-	333	29,43	Membrane protein
lytTR	243	31,28	LytTR family transcriptional regulator
IS431mec	675	34,37	IS6 family transposase IS431mec
-	243	31,28	LytTR family transcriptional regulator
HP	333	29,43	hypothetical protein
