# Supplementary material for: Emergence of Methicillin-Resistant Staphylococcus aureus ST239/241 SCCmec-III Mercury in Eastern Algeria
Source: Pathogens. 2021 Nov 18;10(11):1503. doi: 10.3390/pathogens10111503 (PMC8621676; doi:10.3390/pathogens10111503)
Supplement: Supplementary file 1 [file pathogens-10-01503-s001.zip › Table S4.pdf]

Table S4. Primers and probes used in this study for the molecular characterisation of the clinical *S. aureus* isolates from MICU at UHCC.

Primers or probes	Oligonucleotides (5'-3')	References
**ccrB Type I**		[53]
F_ *ccr*B Type I	TTTGGCACGTAATACTTCCGATT	
R_ *ccr*B Type I	AAAATTCAACATTTTGGCGATGA	
P_ *ccr*B Type I	ACTTACAATAGTCGAAGAAC	
**ccrB Type 1I**		
F_ *ccr*B Type II	AACGAGACGTGCCCAAGAAG	
R_ *ccr*B Type II	CATCAGTTCATGTTTACTATTAGGTATTTTGTC	
P_ *ccr*B Type II	ATTTGCCGCTGGGCT	
**ccrB Type 1II**		
F_ *ccr*B Type III	ACAATCCACAGTCATTACAT	
R_ *ccr*B Type III	AGTTACGACTTTCTGTTTCA	
P_ *ccr*B Type III	ACAAGTCACCGAACGCATCCAACAAA	
**ccrB Type 1V**		
F_ *ccr*B Type IV	GAACAGACCTGAGCTCCAACGT	
R_ *ccr*B Type IV	TCGGTTTGTTTTGTAGATCATAACACA	
P_ *ccr*B Type IV	ATGCAAAAGAAGGCAATAT	
**SCC*mec* V**		[54]
Type V-F	TCTGGGAGTTCTGCCTGTCA	
Type V-R	TCACATTTGACGCAATCTGCT	
Type V-P	TGCTGAAGTCGTCGAACGCGTAATCA	
**SCC*mec* III mercury element**		[14]
Type III-F	CCATATTGTGTACGATGCG	
Type III-R	CCTTAGTTGTCGTAACAGATCG	
Spa		[31]
Spa-1113f	TAAAGACGATCCTTCGGTGAG C	
Spa-1514r	CAGCAGTAGTGCCGTTTGCTT	
**PVL**		[53]
pvl-F	AAAATGCCAGTGTTATCCAGAGGTA	
pvl-R	TTTGCAGCGTTTTGTTTTCG	
pvl-P	CTTCAATCCAGAATTTATTGGTGT	
**TSST-1**		
tsst-1-F	TCATCAGCTAACTCAAATACATGGATT	
tsst-1-R	TGTGGATCCGTCATTCATTGTT	
tsst-1-P	TCCAATAACCACCCGTTTTATCGCTTGAA	
***mec* A**		[54]
*mec*A-F	GTTAGATTGGGATCATAGCGTCATT	
*mec*A-R	TGCCTAATCTCATATGTGTTCCTGTAT	
*mec*A-p	TTCCAGGAATGCAGAAAGACCAAAGCAT	
***mec*C**		[55]
*mec*C-F	GCAAGCAATAGAATCATCAGACAAC	
*mec*C-R	TCTTGCATACCTTGCTCAAATTTT	
*mec*C-P	CCGCATTGCATTAGCATTAGGAGCCA	
